# Mapping Algorithmic Bias in AI-Powered Electrocardiogram Interpretation Across the AI Life Cycle: Protocol for a Scoping Review

**DOI:** 10.2196/82486

**Published:** 2026-01-20

**Authors:** Luqman Lawal, Christopher Paton, Mike English, Bruno Holthof, Tabitha Preston

**Affiliations:** 1 Nuffield Department of Medicine University of Oxford Oxford, England United Kingdom; 2 Mayo Clinic Rochester, MN United States; 3 Liggins Institute and School of Population Health University of Auckland Auckland New Zealand

**Keywords:** algorithmic bias, artificial intelligence, AI, diagnostic fairness, electrocardiography, health equity, machine learning, cardiovascular diagnostics, scoping review

## Abstract

**Background:**

Artificial intelligence (AI)–powered analysis of electrocardiograms (ECGs) is reshaping cardiac diagnostics, offering faster and often more accurate detection of conditions such as arrhythmias and heart failure. However, growing evidence suggests that algorithmic bias, defined as performance disparities across patient subgroups, may undermine diagnostic equity. These biases can emerge at any stage of the AI life cycle, including data collection, model development, evaluation, deployment, and clinical use. If unaddressed, they risk exacerbating health disparities, particularly in underrepresented populations and low-resource settings. Early identification and mitigation of such bias are essential to ensuring diagnostic equity.

**Objective:**

This scoping review protocol outlines a structured approach to mapping the evidence on algorithmic bias in AI-enabled ECG interpretation. Following the population-concept-context framework and PRISMA-ScR (Preferred Reporting Items for Systematic Reviews and Meta-Analyses Extension for Scoping Reviews) guidance, the planned review will systematically identify and categorize reported sources and types of bias, examine their effects on diagnostic performance across demographic and geographic subgroups, and document mitigation strategies applied throughout the AI life cycle. By synthesizing how bias and fairness considerations are handled in this field, this review aims to clarify existing evidence, highlight key gaps, and inform future efforts toward equitable and clinically trustworthy application of AI in cardiology.

**Methods:**

We will conduct a comprehensive literature search across 5 electronic databases (PubMed, Embase, Cochrane CENTRAL, CINAHL, and IEEE Xplore) and gray literature sources. Eligible studies will include original research (2015-2025) evaluating the performance of AI-based ECG models across different subgroups or reporting on bias mitigation strategies. Two reviewers will independently screen studies, extract data using a standardized form, and resolve disagreements through consensus. This review will follow the PRISMA-ScR reporting framework.

**Results:**

At the time of submission, study identification and screening has been completed. Database searches conducted in August and September 2025 yielded 430 records, with an additional 18 records identified through other sources. After duplicates removal, 398 unique records remained. Title and abstract screening led to the exclusion of 250 records, and 148 articles proceeded to full-text review. Following full-text assessment, 110 articles were evaluated for eligibility, of which 38 studies met the inclusion criteria and were included in the qualitative synthesis. The study selection process is summarized in a PRISMA (Preferred Reporting Items for Systematic Reviews and Meta-Analyses) flow diagram. Data extraction was conducted between November and December 2025.

**Conclusions:**

This review will be the first to comprehensively map the landscape of algorithmic bias in AI-powered ECG interpretation. By identifying patterns of inequity and evaluating proposed solutions, it will provide actionable insights for developers, clinicians, and policymakers aiming to promote fairness in AI-enabled cardiac care.

**International Registered Report Identifier (IRRID):**

PRR1-10.2196/82486

## Introduction

### Background

Artificial intelligence (AI)–enabled electrocardiogram (ECG; AI-ECG) interpretation has emerged as a transformative tool in cardiovascular diagnostics. These models, particularly those built using deep learning architectures, have demonstrated high accuracy in identifying cardiac conditions such as atrial fibrillation, heart failure, and left ventricular dysfunction [1,2]. Given the global burden of cardiovascular disease, particularly in low-resource settings, AI-ECG tools offer the potential to enhance access to timely diagnosis, especially in areas with limited cardiology expertise [3].

However, as AI adoption accelerates, concerns about algorithmic bias have gained prominence. Algorithmic bias refers to systematic disparities in model performance across subpopulations, often arising from unrepresentative training data, flawed modeling assumptions, or inappropriate deployment environments [4-6]. These biases can lead to unequal diagnostic accuracy and risk misdiagnosis, particularly in underrepresented or structurally marginalized groups. In cardiology, for instance, tools trained predominantly on datasets composed predominantly of White male individuals have underperformed in detecting myocardial infarction in women and individuals of African descent [7,8]. Physical diagnostic tools such as pulse oximeters have similarly demonstrated racial bias in measurement accuracy [9].

Bias may emerge across multiple stages of the AI development pipeline, from data collection and labeling to model training, evaluation and deployment, and clinical integration. Recent frameworks categorize these into 5 key stages: data, model, evaluation, deployment, and postdeployment bias [10]. Addressing each of these stages is critical to ensuring fairness, safety, and clinical utility. High-income countries (HICs) have started to institutionalize fairness evaluations in medical AI development, with regulatory and ethical guidelines now recommending transparency, community engagement, and subgroup performance monitoring [11-13].

Despite this progress, significant gaps persist in understanding algorithmic bias within AI-ECG models, particularly in low- and middle-income countries (LMICs). Most existing studies and validations are based on datasets from North America and Europe, often excluding the very populations that might benefit most from AI-based screening tools [14]. This mismatch, described as “health data poverty” or “digital colonization,” raises concerns that AI tools exported to LMICs may underperform or cause unintended harm [15,16].

To date, there is no comprehensive synthesis of how bias manifests in AI-powered ECG interpretation. Existing reviews focus primarily on the diagnostic accuracy of AI-ECG for specific conditions and seldom report performance stratified by demographic or geographic subgroups [17]. A few studies have started to explore fairness or subgroup performance. However, findings are scattered, and no previous scoping review has mapped the full range of bias-related evidence or mitigation efforts in this field. It remains unclear which populations are most affected, at what stages bias arises, and what strategies have been attempted to reduce disparities.

With the increasing deployment of AI-ECG tools, including in LMICs, there is an urgent need to map the evidence on algorithmic bias in this domain systematically. Doing so will support the equitable development and safe deployment of AI-based ECG technologies across diverse populations and health systems. Early identification and mitigation of such bias are essential to diagnostic equity.

### Objectives

This scoping review aims to comprehensively map and characterize the current evidence related to algorithmic bias in AI-powered ECG interpretation across the AI life cycle.

This review is guided by the population-concept-context (PCC) framework, which also informs the eligibility criteria:

- Population—adult patients (≥18 years) undergoing ECG-based cardiac assessment, particularly those from underrepresented groups (eg, women, racial and ethnic minority groups, and LMIC populations)
- Concept—algorithmic bias in AI-powered ECG interpretation, including disparities in diagnostic accuracy, error rates, and subgroup performance as well as documented mitigation strategies
- Context—any health care setting where AI-ECG models are developed, validated, or deployed, including both HICs and LMICs.

By addressing the following research questions, this review aims to provide a comprehensive overview of the field:

1. What types of algorithmic bias are reported in AI-powered ECG interpretation models?
2. At which stages of the AI life cycle (data, model, evaluation, deployment, and postdeployment stage) do these biases occur?
3. How do identified biases affect diagnostic performance and health care equity across different patient groups?
4. What mitigation strategies or fairness interventions have been proposed or tested in the context of AI-ECG?
5. How does AI-ECG performance vary across demographic and geographic subgroups, including sex and gender, race and ethnicity, age, and LMIC versus HIC settings?

This scoping review will inform future research, regulation, and implementation of AI-based cardiac diagnostics to ensure they serve all populations equitably.

## Methods

### Protocol Design

This study will be conducted as a scoping review, following the framework by Arksey and O’Malley [18], with enhancements by Levac et al [19] and methodological guidance from the Joanna Briggs Institute [20]. This review will be reported following the PRISMA-ScR (Preferred Reporting Items for Systematic Reviews and Meta-Analyses Extension for Scoping Reviews) checklist [21].

We will undertake the five standard stages of scoping reviews: (1) identifying the research question; (2) identifying relevant studies; (3) selecting studies; (4) charting the data; and (5) collating, summarizing, and reporting results.

We will skip the optional sixth stage (stakeholder consultation) because of resource and timing constraints.

### Eligibility Criteria

Eligibility criteria are defined using the PCC framework recommended for scoping reviews.

#### Population

We will include studies involving adult human participants (aged ≥18 y) whose ECG data were analyzed using an AI-based tool. Studies must report subgroup performance data (eg, by sex, race and ethnicity, age, or geographic setting), even if they do not explicitly mention “bias.” Pediatric populations will be excluded.

#### Concept

The phenomenon of interest is algorithmic bias in AI-powered ECG interpretation. Eligible studies must evaluate AI models (eg, machine learning and deep learning) used to interpret ECGs for cardiac diagnosis and must report on performance disparities, fairness metrics, or generalizability. Both commercial and research models will be included. Studies that assess model performance across groups, whether or not they use fairness terminology, will be eligible.

#### Context

We will include studies conducted in any global health care or research setting, including HICs and LMICs, across any level of care. No restrictions will be placed on the study setting or geographic region.

#### Study Designs

Eligible sources include all empirical studies presenting original data on AI-ECG performance and bias. These include diagnostic accuracy studies, randomized controlled trials, cohort and case-control studies, cross-sectional and retrospective validation studies, and technical validation studies (eg, internal or external validations of AI algorithms).

We will also include preprints, conference abstracts, and dissertations. Excluded sources include narrative and systematic reviews, commentaries, editorials, and purely theoretical or methodological papers without patient-level data.

#### Comparators

Studies with explicit or implicit comparators (eg, comparing model outputs across subgroups or against clinicians) will be included. No comparator is required if subgroup-specific performance metrics are reported.

#### Time Frame

Studies published from January 1, 2015, to July 31, 2025, will be eligible. This window reflects the period during which deep learning models became prominent in ECG interpretation [22].

#### Language

Only studies published in English were included. We acknowledge this as a limitation and will report any potentially relevant non-English studies excluded on this basis.

### Information Sources and Search Strategy

#### Search Strategy Development

A comprehensive search strategy will be developed in collaboration with a medical librarian and peer reviewed using the Peer Review of Electronic Search Strategies (PRESS) checklist [23]. We will use both controlled vocabulary and free-text terms reflecting the PCC elements.

#### Databases

We will search the following databases including MEDLINE (via PubMed), Embase via Ovid, Cochrane CENTRAL, CINAHL (EBSCO), IEEE Xplore, and Web of Science Core Collection (as supplemental sources).

#### Gray Literature

Additional sources to be assessed include ProQuest Dissertations and Theses.

To optimize resource use while ensuring comprehensiveness, we will limit gray literature inclusion to a small set of high-yield sources. Specifically, we will search for conference abstracts from 3 major conferences where AI and ECG research is typically presented: American Heart Association, European Society of Cardiology, and American Medical Informatics Association.

We will limit the search to the years 2020 to 2025, using keyword combinations related to “ECG,” “artificial intelligence,” and “bias” in the title and abstract fields. Only abstracts that report original development or evaluation of AI-ECG tools will be included.

We will not include trial registries (eg, World Health Organization International Clinical Trials Registry Platform) because most entries lack completed results or sufficient methodological detail, and we aim to prioritize sources that offer extractable insights into model bias and evaluation.

Each step in the search and screening process will be documented, with independent screening by 2 reviewers to ensure transparency and reproducibility.

#### Search Terms

Search strings will include terms such as follows: (“Artificial Intelligence” OR “Machine Learning” OR “Deep Learning”) AND (“ECG” OR “Electrocardiogram”) AND (“Bias” OR “Fairness” OR “Health Equity” OR “Disparities” OR “Subgroup Analysis”).

Search strategies will be customized for each database. Full strategies will be provided in Multimedia Appendix 1. Filters will be applied for publication date (2015-2025) and English language.

To ensure sensitivity to global equity considerations, the primary search strategy will remain unrestricted by income-level terminology, preventing unintentional exclusion of studies that include LMIC data but do not explicitly use LMIC descriptors.

During data extraction, we will record geographic context and setting (LMIC, HIC, mixed, or unspecified) to support structured equity analysis. A supplementary targeted search using LMIC-related terms (eg, “low- and middle-income countries” and “resource-limited settings”) and citation chaining will be conducted to increase the likelihood of capturing LMIC-specific literature.

### Study Selection

#### Screening Procedure

Study selection will occur in 2 stages: title and abstract screening and full-text screening.

Two reviewers will screen independently. Discrepancies will be resolved by consensus or by a third reviewer. A pilot of 100 citations will calibrate interreviewer agreement.

#### Screening Tool

Covidence (Veritas Health Innovation) will be used to manage the screening process and document inclusion and exclusion decisions.

#### PRISMA-ScR Flow Diagram

Study selection will be reported using a PRISMA-ScR flowchart, including reasons for exclusion at the full-text stage.

### Data Extraction (Charting the Data)

#### Data Extraction Process

A structured data extraction form will be developed and piloted. Two reviewers will independently extract data from a sample of studies; one reviewer will complete the full extraction, with a second reviewer verifying all entries.

To ensure consistent interpretation of bias across studies, we will use a hierarchical extraction framework with predefined decision rules for each bias domain (sampling, measurement, label, aggregation, evaluation, and deployment). Reviewers will record (1) explicitly reported bias, (2) implicit evidence of bias (eg, subgroup performance differences and nonrepresentative datasets), or (3) insufficient information. Each domain is linked to operational indicators adapted from established AI ethics taxonomies [9], as shown in Multimedia Appendix 1. Two reviewers will independently extract and code bias-related information, with discrepancies resolved through consensus or adjudication by a third reviewer. This structured approach supports transparency and reproducibility in identifying bias manifestation across the AI life cycle.

#### Data Items

Key variables to be extracted are presented in Textbox 1 [9].

**Data items extracted in this scoping review.**

- Study characteristics will include the author, year of publication, country, and journal or source.
- Study design and setting will include the study type and context, including whether the study was conducted in high-income countries or low- and middle-income countries and in primary or tertiary care settings.
- Population characteristics will include sample size, age, sex, race and ethnicity, and reported comorbidities.
- Artificial intelligence model characteristics will include the algorithm type, clinical task, development datasets, and model status (research or commercial).
- Performance and bias findings will include stratified performance metrics (eg, area under the curve, sensitivity, and specificity), subgroup disparities, and statistical significance.
- Bias life cycle stage will be recorded as data, model, evaluation, deployment, or postdeployment stage, classified per the typology by Mehrabi et al [9]. Operational definitions, decision criteria, and exemplar indicators for each bias category will guide reviewer judgment and ensure consistent application of the coding framework.
- Mitigation strategies will include techniques such as resampling, fairness constraints, post hoc calibration, or external validation.
- Author conclusions and implications will be extracted as reported in the included studies.

Data will be stored in Microsoft Excel or equivalent software for analysis. Ethical and contextual domains, including fairness reporting, dataset origin, governance, and community engagement, will be systematically extracted as dedicated fields to evaluate risks of digital colonization and data inequity.

Furthermore, repeated use of canonical ECG datasets may inflate the perceived generalizability. Such overlap will be treated as a data-stage bias signal, specifically related to sampling and aggregation bias. Studies leveraging the same datasets will be clustered to ensure findings are not interpreted as independent replications.

### Data Analysis and Synthesis

#### Synthesis Approach

We will use descriptive and thematic analysis. Results will be synthesized to address the 5 guiding questions of the review as stated in the *Objective* section. Quantitative summaries will describe study counts, subgroup types, and bias stages. Narrative synthesis will highlight bias types and life cycle stages, performance disparities across subgroups, effectiveness of mitigation strategies, and geographic and contextual representation (HIC vs LMIC).

#### Visualizations

Findings will be presented through evidence tables (eg, bias type by study), charts or maps (eg, study distribution by year or geography), and summary matrices (eg, subgroup performance data).

#### Gaps and Recommendations

We will identify evidence gaps (eg, lack of LMIC data and underexplored bias stages) and recommend future research priorities. No meta-analysis will be performed due to expected heterogeneity.

### Quality Appraisal

Consistent with Joanna Briggs Institute and PRISMA-ScR guidance, we will not conduct a formal quality appraisal. However, we will document evident methodological limitations (eg, small sample sizes or limited subgroup analysis) that may affect interpretation.

In studies reporting AI model development or validation, we will extract selected bias-related indicators aligned with the Prediction model Risk of Bias Assessment Tool–AI framework [24]. Although full risk-of-bias scoring will not be performed, this approach will support structured identification of potential bias-related to participant selection, data quality, model evaluation, and fairness reporting and will help flag limitations affecting subgroup generalizability.

### Ethical Considerations

This review involves secondary analysis of publicly available literature and does not include any original research involving human participants. Therefore, it does not require approval from an institutional review board or ethics committee. No individual-level, sensitive, or identifiable data will be accessed or analyzed during this study, consistent with established ethical standards for systematic reviews [23].

### Dissemination

The results of this scoping review will be submitted for publication in a peer-reviewed journal focused on digital health, AI in medicine, or cardiovascular care. In addition to traditional academic dissemination through journal articles and conference presentations, findings will also be shared with key stakeholders, such as health care AI developers, clinicians, and policymakers, via webinars, social media, and professional networks. Special emphasis will be placed on sharing the findings in ways that are accessible to stakeholders in LMICs, where bias in AI tools can be particularly impactful.

## Results

### Timeline

**Figure 1:**
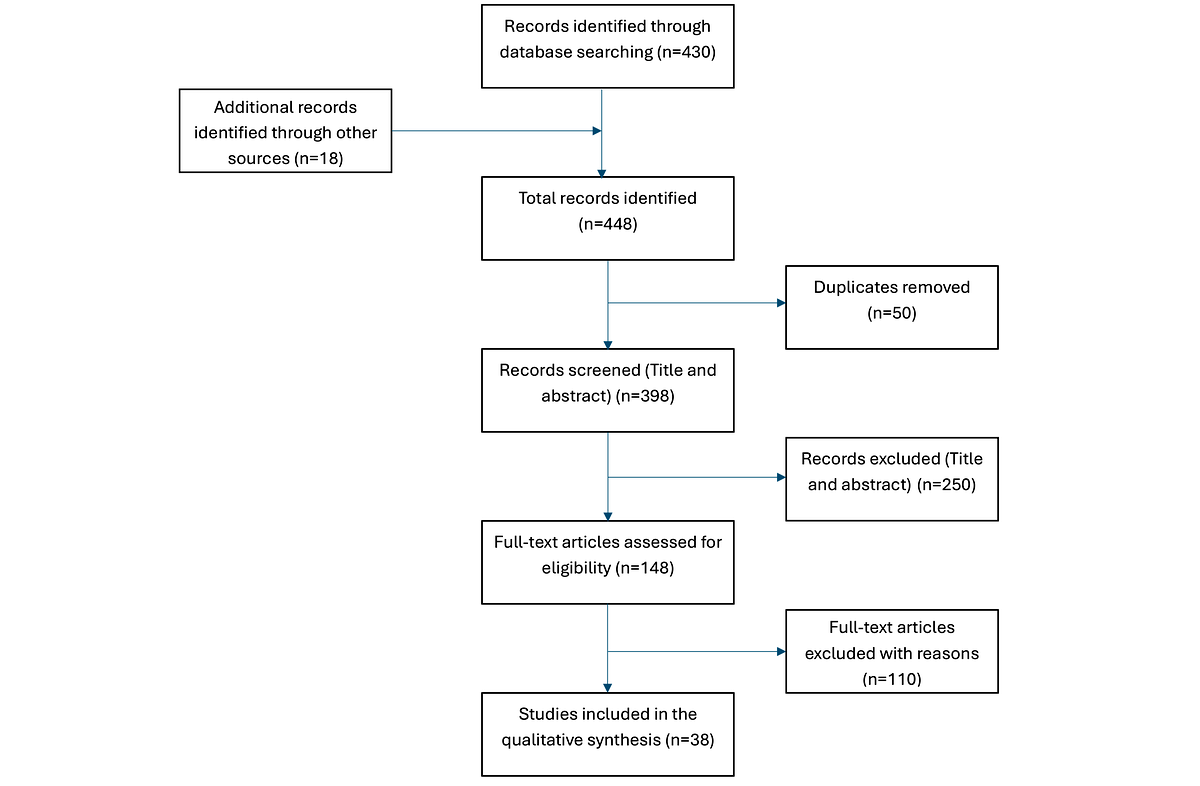
PRISMA (Preferred Reporting Items for Systematic Reviews and Meta-Analyses) flowchart.

The database search was conducted in August and September 2025. As displayed in Figure 1, study screening and data extraction took place from November 2025 to December 2025. Data analysis and manuscript submission are planned for January and February 2026.

### Reporting Standards Compliance

This protocol is reported following the PRISMA-P (Preferred Reporting Items for Systematic Review and Meta-Analyses Protocols) 2015 guidelines.

### Progress to Date

The literature search strategy was finalized and executed across the specified databases in August and September 2025. Following duplicate removal, title and abstract screening was completed by two independent reviewers, leading to the identification of 148 records for full-text review. The full-text assessment concluded in November 2025, and 38 studies met the inclusion criteria for the qualitative synthesis. Data extraction was conducted using a standardized charting form between November and December 2025, with all extracted entries verified by a second reviewer. Descriptive and thematic analysis of the collated evidence is currently underway, with completion targeted for February 2026. The study selection process is summarized in a PRISMA flow diagram (Figure 1).

### Anticipated Completion

We plan to complete the main scoping review manuscript by the end of February 2026. This will include final synthesis of extracted data, preparation of summary evidence tables and bias classification matrices, and drafting of the Results and Discussion sections. The completed review manuscript will be submitted for peer-reviewed publication shortly thereafter, consistent with PRISMA-ScR guidance and journal requirements.

## Discussion

This protocol outlines a robust and comprehensive approach to mapping the evidence on algorithmic bias in AI-powered ECG interpretation. By applying the PCC framework and PRISMA-ScR guidance, this study is designed to identify and categorize systematically reported biases, assess their impact on diagnostic performance, and review mitigation strategies across the AI life cycle.

One of the main anticipated contributions of this review is its focus on both HIC and LMIC contexts, where the implications of algorithmic bias may differ substantially due to variations in data availability, clinical infrastructure, and deployment conditions. In doing so, this review addresses the persistent gap in the global representativeness of AI-ECG research and the underreporting of subgroup performance metrics.

This work is timely given the accelerating integration of AI in cardiovascular diagnostics and the increasing recognition that unchecked bias can exacerbate health inequities. Although previous reviews have examined the accuracy of AI-ECG systems, none have comprehensively mapped evidence on bias manifestations and mitigation efforts. By documenting both the problem space and potential solutions, our findings will provide actionable insights for developers, regulators, and clinicians.

We acknowledge certain limitations inherent to our protocol. Restricting inclusion to English-language publications may omit relevant studies, particularly from non–English-speaking LMICs. The expected heterogeneity in study designs and reporting standards will preclude meta-analysis, limiting the synthesis to descriptive and narrative methods. In addition, the focus on published and indexed gray literature means that some proprietary or unpublished industry data, where bias assessments may have been conducted, will remain inaccessible.

Despite these constraints, our methodology incorporates multiple strategies to ensure comprehensiveness and transparency, including a librarian-led search strategy, independent screening by 2 reviewers, and standardized data extraction. The planned descriptive and thematic synthesis will enable a nuanced understanding of bias types, affected populations, and life cycle stages as well as the relative maturity of mitigation strategies.

Ultimately, the outputs of this review will serve as an evidence base for equitable AI-ECG development, deployment, and governance, helping to ensure that technological advancements in cardiology benefit all patient populations.

## Data Availability

The dataset generated from this scoping review will consist of extracted information from published studies. The extracted data will be made available from the corresponding author upon reasonable request after publication, subject to journal policies and any applicable restrictions.

## Appendix

Multimedia Appendix 1 Detailed database search strategies, eligibility criteria, and study selection framework based on the population–concept–context approach, data extraction template and variable definitions, bias classification framework across the artificial intelligence life cycle, operational definitions and coding rules for bias assessment, and summary of the extracted study characteristics and analytical fields.

## Abbreviations

AI: artificial intelligence
AI-ECG: artificial intelligence–enabled electrocardiogram
ECG: electrocardiogram
HIC: high-income country
LMIC: low- and middle-income country
PRESS: Peer Review of Electronic Search Strategies
PRISMA-P: Preferred Reporting Items for Systematic Review and Meta-Analyses Protocols
PRISMA-ScR: Preferred Reporting Items for Systematic Reviews and Meta-Analyses Extension for Scoping Reviews
