# Supplementary material for: Mapping Algorithmic Bias in AI-Powered Electrocardiogram Interpretation Across the AI Life Cycle: Protocol for a Scoping Review
Source: JMIR Res Protoc. 2026 Jan 20;15:e82486. doi: 10.2196/82486 (PMC12869145; doi:10.2196/82486)
Supplement: Multimedia Appendix 1 [file resprot_v15i1e82486_app1.docx]

## 1. Database Search Strings

*Tab 1*

| **Database** | **Search String** |
| --- | --- |
| MEDLINE / PubMed | (("Artificial Intelligence"[Mesh] OR "Machine Learning"[Mesh] OR "Deep Learning"[Mesh]  OR "artificial intelligence"[tiab] OR "machine learning"[tiab] OR "deep learning"[tiab]  OR "neural network*"[tiab] OR algorithm*[tiab])  AND ("Electrocardiography"[Mesh] OR "Electrocardiography, Ambulatory"[Mesh]  OR "ECG"[tiab] OR "EKG"[tiab] OR "electrocardiogram*"[tiab])  AND ("Health Equity"[Mesh] OR "Healthcare Disparities"[Mesh] OR "Bias"[Mesh] OR "Prejudice"[Mesh]  OR fairness[tiab] OR "health equity"[tiab] OR disparit*[tiab]  OR "subgroup analy*"[tiab] OR generalizability[tiab] OR "performance variation"[tiab]))  AND ("2015/01/01"[Date - Publication] : "3000"[Date - Publication])  AND English[lang] |
| EMBASE (Ovid) | (('artificial intelligence'/exp OR 'machine learning'/exp OR 'deep learning'/exp  OR 'artificial intelligence':ti,ab OR 'machine learning':ti,ab OR 'deep learning':ti,ab  OR 'neural network*':ti,ab OR algorithm*:ti,ab)  AND ('electrocardiography'/exp OR ECG:ti,ab OR EKG:ti,ab OR electrocardiogram*:ti,ab)  AND ('health equity'/exp OR 'health care disparity'/exp OR 'bias'/exp OR 'prejudice'/exp  OR fairness:ti,ab OR 'health equity':ti,ab OR disparit*:ti,ab  OR 'subgroup analy*':ti,ab OR generalizability:ti,ab OR 'performance variation':ti,ab))  AND [english]/lim AND [2015-2025]/py |
| CINAHL (EBSCO) | ((MH "Artificial Intelligence+") OR (MH "Machine Learning+") OR (MH "Deep Learning+")  OR TI,AB("artificial intelligence" OR "machine learning" OR "deep learning" OR "neural network*" OR algorithm*))  AND ((MH "Electrocardiography+") OR TI,AB("ECG" OR "EKG" OR "electrocardiogram*"))  AND ((MH "Health Equity+") OR (MH "Healthcare Disparities+") OR (MH "Bias+") OR (MH "Prejudice+")  OR TI,AB(fairness OR "health equity" OR disparit* OR "subgroup analy*" OR generalizability OR "performance variation")))  AND (LA English) AND (DT 20150101-20251231) |
| IEEE Xplore | (("artificial intelligence" OR "machine learning" OR "deep learning"  OR "neural network*" OR algorithm*)  AND ("ECG" OR "EKG" OR "electrocardiogram*")  AND (fairness OR "health equity" OR disparit* OR "subgroup analy*" OR generalizability OR "performance variation"))  AND (Publication Year: 2015-2025) |
| Web of Science (Core Collection) | TS=("artificial intelligence" OR "machine learning" OR "deep learning" OR "neural network*" OR algorithm*)  AND TS=("ECG" OR "EKG" OR "electrocardiogram*")  AND TS=(fairness OR "health equity" OR disparit* OR "subgroup analy*" OR generalizability OR "performance variation")  Refined by: Languages=(ENGLISH) AND Publication Years=(2015-2025) |

## 2. Data Extraction Template

| **Variable** | **Definition / Coding Instructions** |
| --- | --- |
| Study ID | Unique identifier assigned after screening. |
| Citation | Author(s), year, journal/conference, DOI. |
| Country / Region | Country where the study was conducted or dataset originated. |
| Setting | Clinical setting (e.g., tertiary hospital, outpatient clinic, community health center). |
| Income Level | World Bank classification of country (HIC, UMIC, LMIC, LIC). |
| Study Design | RCT, observational, case-control, diagnostic accuracy, validation, simulation, etc. |
| AI Model Type | Architecture (e.g., CNN, RNN, hybrid), proprietary vs. open-source. |
| Training Data Source | Dataset name, origin, size, and population characteristics. |
| Test Data Source | Dataset name, origin, size, and population characteristics. |
| Bias Type | Using Mehrabi et al.’s typology (sampling bias, measurement bias, etc.). |
| AI Lifecycle Stage | Stage(s) where bias was reported (data collection, preprocessing, modeling, deployment). |
| Performance Metrics Reported | AUROC, sensitivity, specificity, PPV, NPV, F1, etc. |
| Subgroup Analyses | Whether performance metrics were stratified by demographics or comorbidities. |
| Mitigation Strategies | Bias mitigation approaches described (e.g., data augmentation, reweighting). |
| Regulatory / Ethical Considerations | Any discussion of fairness, equity, or compliance with standards. |
| Key Findings | Summary of main results related to bias. |
| Limitations Reported | Limitations acknowledged by the authors regarding bias. |
| Reviewer Notes | Free-text space for reviewer comments. |

*Tab 2*

### 3. PRISMA -ScR Checklist Compliance Table for Systematic Review Protocol

| Section | Item No. | Checklist Item | Location in Protocol |
| --- | --- | --- | --- |
| TITLE | 1 | Identify the report as a scoping review. | 1 |
| Abstract | 2 | Provide a structured summary including background, objectives, eligibility criteria, sources of evidence, charting methods, results, and conclusions that relate to the review questions and objectives. | 3, 4 |
| Introduction | 3 | Describe the rationale for the review in the context of what is already known. | 5 |
|  | 4 | Provide an explicit statement of the objectives or questions the review addresses. | 6 |
| Methods | 5 | Indicate whether a review protocol exists, if and where it can be accessed | 7 |
|  | 6 | Specify characteristics of the sources of evidence (e.g., years considered, language, and publication status) and eligibility criteria used for the review. | 8 |
|  | 7 | Describe all information sources (e.g., databases with dates of coverage, contact with authors) in the search and date last searched. | 9 |
|  | 8 | Present the full electronic search strategy for at least one database, including any limits used, so it could be repeated. | 9 |
|  | 9 | State the process for selecting sources of evidence (screening and eligibility). | 9, 10 |
|  | 10 | Describe the methods of charting data from the included sources (data extraction) and any processes for obtaining or confirming data from investigators. | 10 |
|  | 11 | List and define all variables for which data were sought and any assumptions or simplifications made. | 10 |
|  | 12 | Describe the methods used for critical appraisal of individual sources of evidence (if done), including how this information is to be used in any data synthesis. | 11 |
|  | 13 | Describe the methods of handling and summarizing the data that were charted. | 11 |
| Results | 14 | Give numbers of sources of evidence screened, assessed for eligibility, and included in the review, with reasons for exclusions at each stage, ideally using a flow diagram. | 12 |
|  | 15 | For each source of evidence, present characteristics for which data were charted and provide the citations. | 12 |
|  | 16 | Present the results of any critical appraisal of individual sources of evidence (if done). | 12 |
|  | 17 | Present relevant data that were charted that relate to the review questions and objectives. | 12 |
|  | 18 | Summarize and/or present the results of the synthesis. | 12 |
| Discussion | 19 | Summarize the main results (including an overview of concepts, themes, and types of evidence available), link to the review questions and objectives, and consider the relevance to key groups. | 12 |
|  | 20 | Discuss the limitations of the scoping review process. | 13 |
|  | 21 | Provide a general interpretation of the results with respect to the review questions and objectives, as well as potential implications and/or next steps. | 13 |
| Funding | 22 | Describe sources of funding for the included sources of evidence and for the scoping review itself. Describe the role of the funders. | 14 |

*Tab 3*

### 4. Bias Lifecycle Stage Framework

| Bias Type (Mehrabi et al.) | Operational Definition in This Review | Lifecycle Stage |
| --- | --- | --- |
| Sampling Bias | Systematic differences in patient populations included in the dataset vs. target population. | Data Collection |
| Measurement Bias | Errors in ECG acquisition or labeling that disproportionately affect subgroups. | Data Collection / Preprocessing |
| Label Bias | Inaccuracies or inconsistencies in ground truth (e.g., cardiologist interpretation differences). | Data Annotation |
| Aggregation Bias | Ignoring relevant subgroup differences during modeling, leading to one-size-fits-all models. | Modeling |
| Evaluation Bias | Incomplete or skewed validation datasets that do not represent target population diversity. | Model Evaluation |
| Deployment Bias | Performance degradation or inequity during real-world use due to context differences. | Deployment |

*Tab 4*

### 5. Definition of Terminologies

| **Term** | **Definition** |
| --- | --- |
| Algorithmic Bias | Systematic and unfair performance differences of an AI model between different subgroups of patients. |
| Fairness | The principle of achieving equitable diagnostic performance across patient subgroups. |
| AI Lifecycle Stage | Phases from data collection to deployment, as defined by model development workflows. |
| ECG Interpretation | The process of analyzing electrocardiogram data to detect cardiac conditions. |
|  |  |

*Tab 5*

### 6. Bias Coding Framework

| Bias Type | Operational Indicator | Example Evidence | Reviewer Code |
| --- | --- | --- | --- |
| Sampling | Dataset not representative of target population | Disproportionate % from one region/group | Explicit / Implicit / Not reported |
| Measurement | Signal or feature extraction varies by subgroup | Noisy ECG in women/elderly affects model | Explicit / Implicit / Not reported |
| Label | Inconsistent expert labeling or ground truth bias | Specialist bias in ECG labeling | Explicit / Implicit / Not reported |
| Aggregation | Homogeneous assumptions across heterogeneous groups | Single-population model applied broadly | Explicit / Implicit / Not reported |
| Evaluation | No subgroup stratification in validation | No sex- or ethnicity-specific metrics | Explicit / Implicit / Not reported |
| Deployment | Model underperforms in real-world setting | Performance drop in LMIC or rural sites | Explicit / Implicit / Not reported |

*Tab 6*
